# Supplementary material for: Myosin turnover controls actomyosin contractile instability
Source: Proc Natl Acad Sci U S A. 2022 Oct 20;119(43):e2211431119. doi: 10.1073/pnas.2211431119 (PMC9618044; doi:10.1073/pnas.2211431119)
Supplement: Supplementary File [file pnas.2211431119.sapp.pdf]

PNAS
www.pnas.org

Supplementary Information for

Myosin turnover controls actomyosin contractile instability

Sathish Thiyagarajan[1,2], Shuyuan Wang[1], Ting Gang Chew[3], Junqi Huang[3], Lokesh Kumar[3], Mohan K. Balasubramanian[3], and Ben O'Shaughnessy[2]

[1]Department of Physics, Columbia University, New York, NY, USA

[2]Department of Chemical Engineering, Columbia University, New York, NY, USA

[3]Warwick Medical School, University of Warwick, Coventry, UK

Ben O'Shaughnessy

Email: bo8@columbia.edu

This PDF file includes:

- Figures S1 to S6
- Table S1
- Legend for Movie S1
- SI References

Other supplementary materials for this manuscript include the following:

- Movie S1

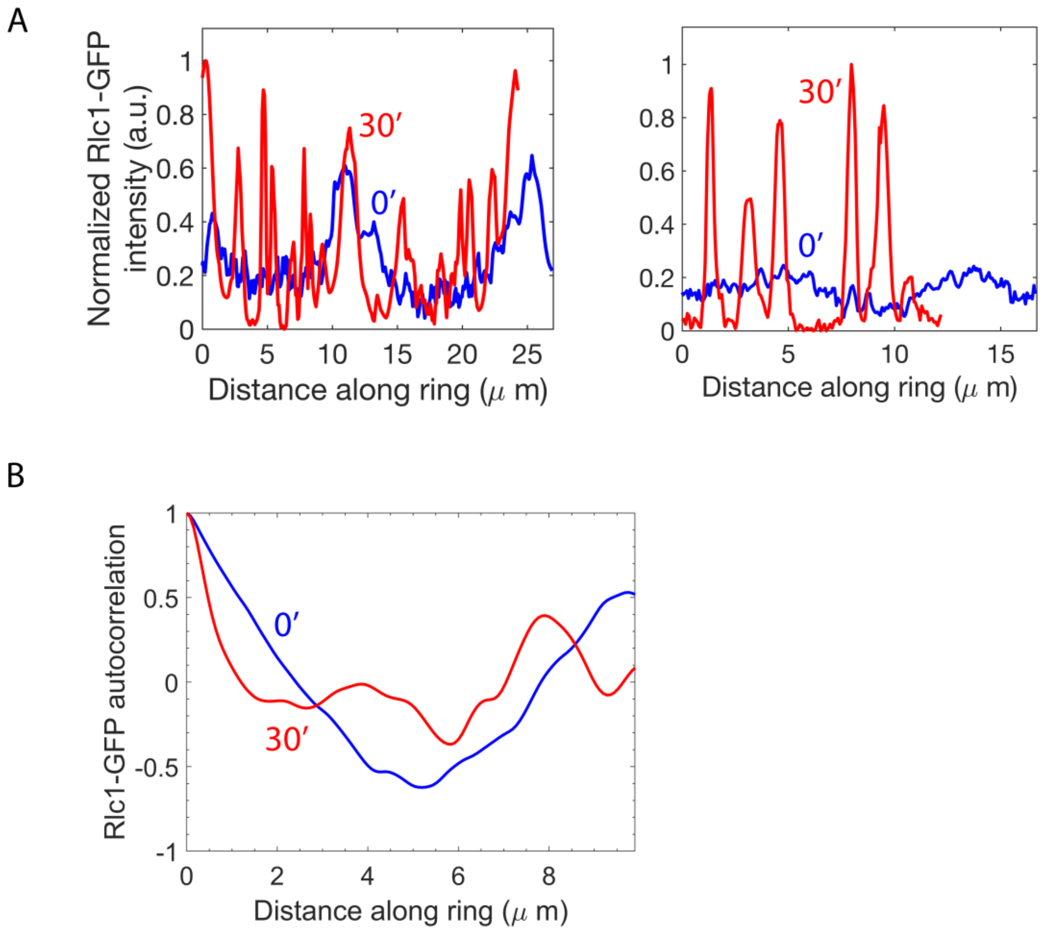

Fig. S1. Related to Rlc1-GFP intensity measurements from Figs. 1 and 2. (A) Rlc1-GFP intensity profiles at the indicated times for the partially unanchored ring (left) and the partially unanchored and severed ring (right) of Fig. 1C. Intensity normalized by the total intensity around the ring at each time. Both profiles extend around the entire ring, including the unanchored and (for the severed ring) the severed portions. (B) Normalized Rlc1-GFP spatial correlations of Fig. 2D at the indicated times.

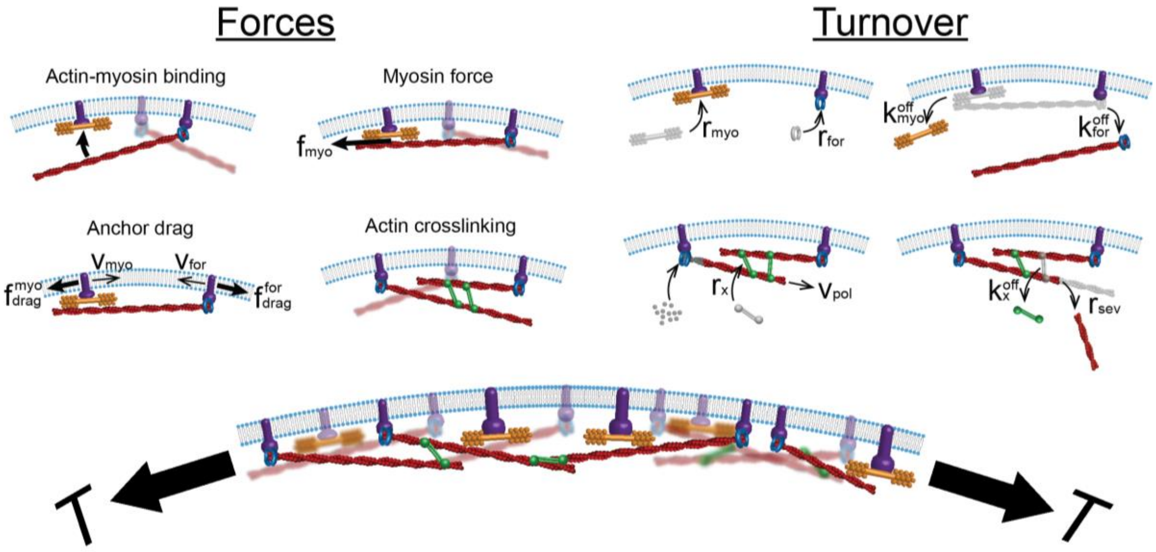

Fig. S2. 3D model of the cytokinetic ring in fission yeast protoplasts (components not to scale). See Table S1 for component amounts and key parameters. Actin filaments are anchored to the plasma membrane at barbed ends by formin Cdc12 and bind membrane-anchored myosin-II that pulls them according to a linear force-velocity relation. Component motions are resisted by drag from the cytoplasm and the plasma membrane (on anchors only). These components constantly turnover: actin is polymerized by formin Cdc12 dimers, dissociates by unbinding with formins, and by stochastic cofilin-mediated filament severing; myosin-II clusters, formin Cdc12 dimers and α-actinin crosslinks constantly bind and unbind the ring. In cell ghosts, components no longer bind the ring, and unbinding dynamics are slowed down (see Table S1).

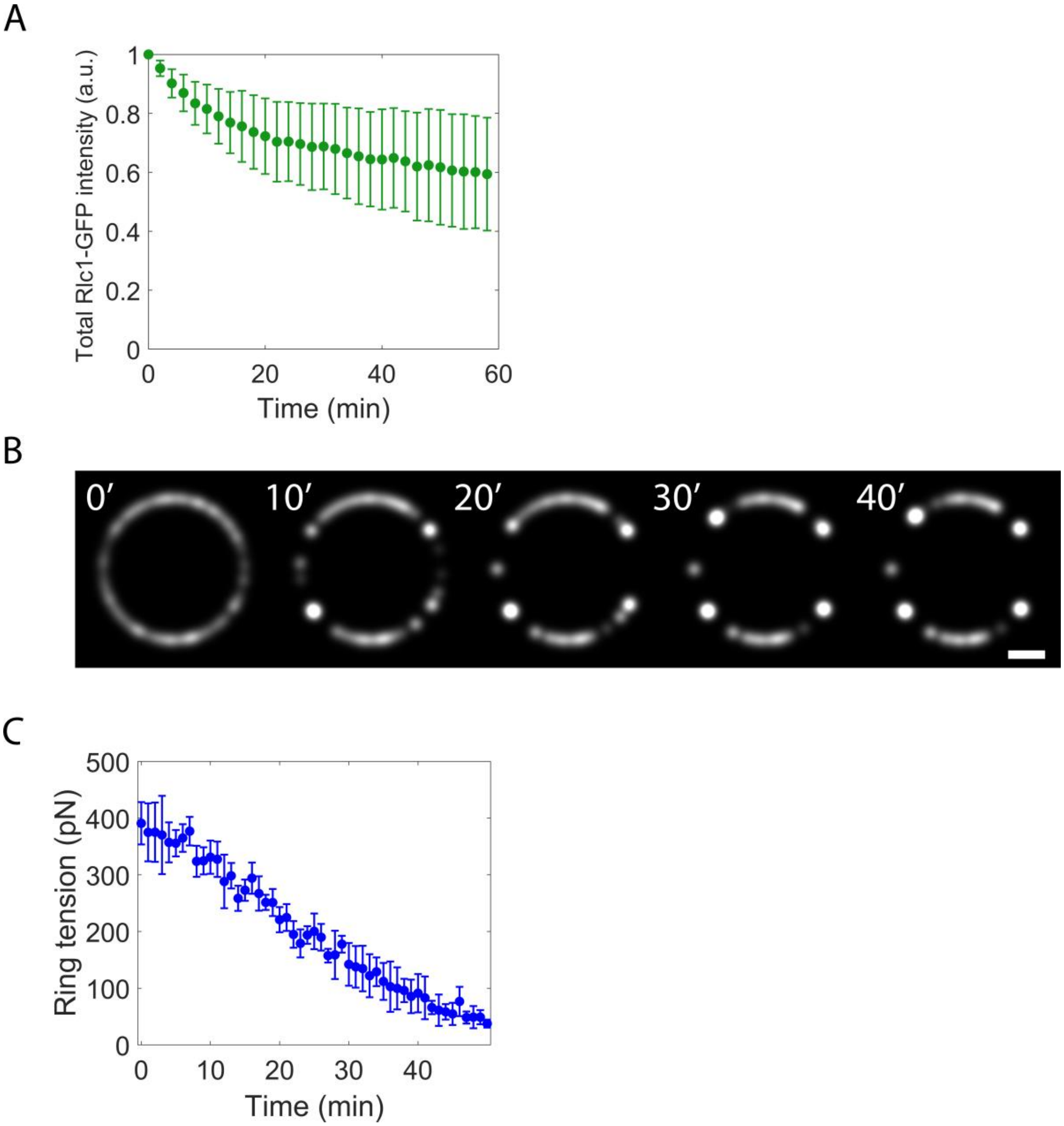

Fig. S3. Myosin aggregation in simulations with no myosin dissociation or with actin turnover but no myosin turnover. (A) Mean total Rlc1-GFP fluorescence intensity along the ring versus time, averaged over the rings of Fig. 2B. Error bars indicate s.d. (B) Simulated confocal fluorescence images of myosin-II distributions from a simulation of the model with no myosin loss after abolition of turnover. Figure preparation and simulation protocol similar to that used in Fig. 3B. Scale bar: 2 μm. (C) Mean ring tension versus time averaged over simulated rings with only actin turnover restored (*n* = 5 rings, error bars are s.d.). ATP is added at time zero.

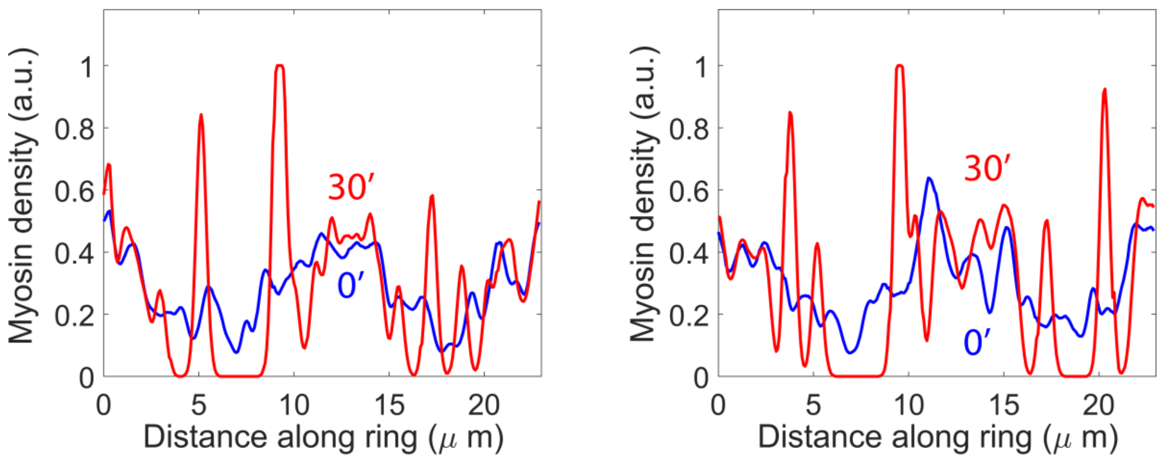

Fig. S4. Relative myosin density versus distance around two simulated rings from the dataset of *n* = 10 rings at the indicated times. Each curve was normalized by total amount of myosin at the respective time.

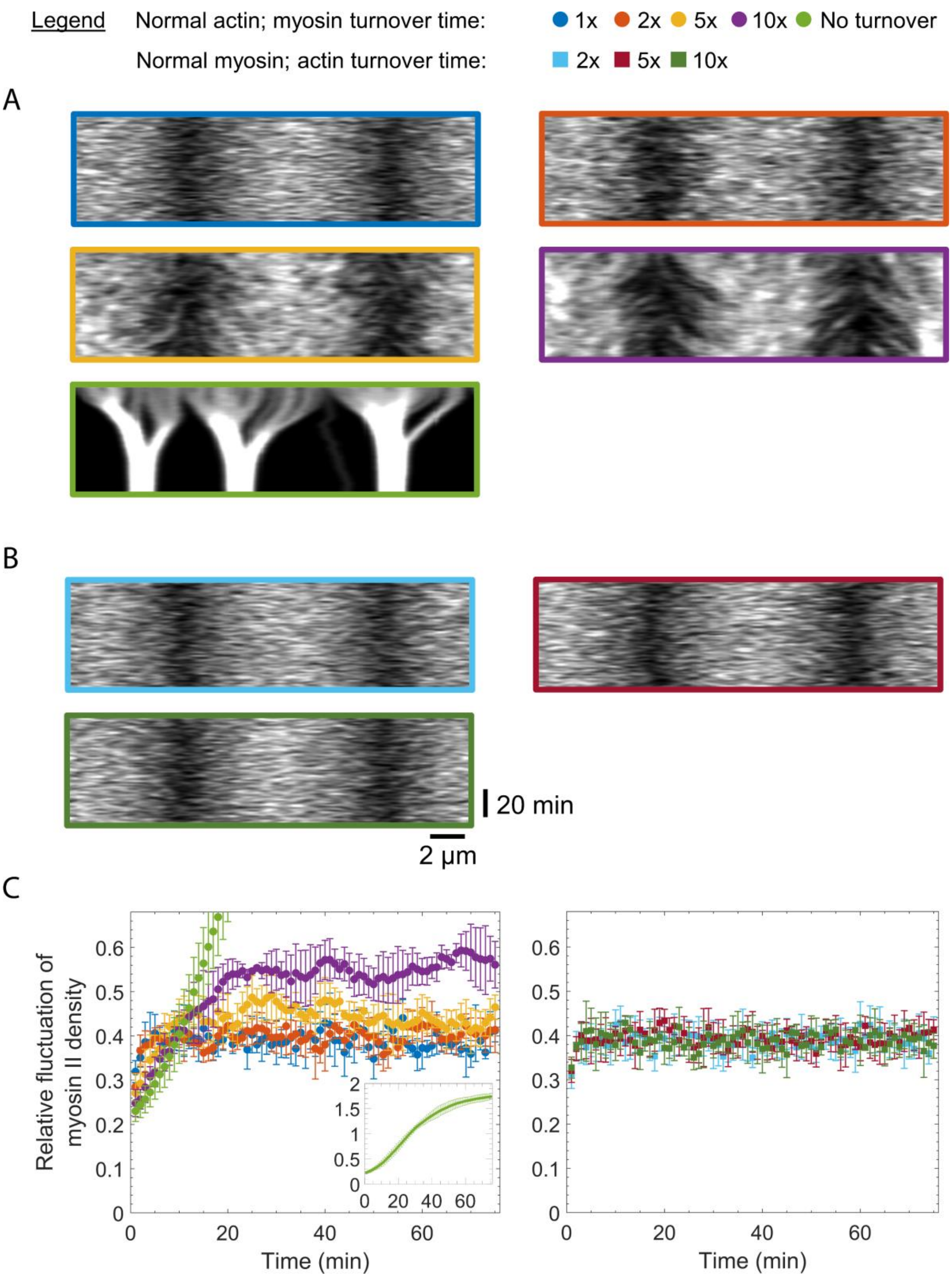

Fig. S5. In simulations with variable myosin and actin turnover times, myosin aggregation effects are manifested for myosin turnover times ~5-fold the normal value or greater. Results from simulated cell ghost rings with variable turnover times as

described in section 7 of Methods. (A) Kymographs from simulated confocal fluorescence images of myosin II with myosin turnover slower than in normal intact cells and with variable turnover times (see legend), while turnover of actin is unchanged from the value in normal intact cells. The first signs of myosin aggregation are apparent when myosin turnover is ~ 5-fold slower than normal. (B) Kymographs from simulated confocal fluorescence images of myosin with myosin turnover as in normal intact cells, but with actin turnover slower than in normal cells and with variable turnover times (see legend). (C) Relative myosin density fluctuations (ratio of s.d. to mean) versus time, measured from kymographs generated by simulations, with various fold increases of the turnover time of myosin or of actin relative to normal cells, see legend (n = 5 runs per turnover condition, values are mean ± s.d.). Left (right) panel shows fluctuations for myosin (actin) turnover times greater than in normal cells. Inset of left panel: fluctuations versus time for normal actin turnover and no myosin turnover. For myosin turnover at least ~ 5-fold slower than normal, myosin fluctuations are larger than normal, an indication of aggregation. The spatial and temporal scales for (A) and (B) are the same (see scale bars).

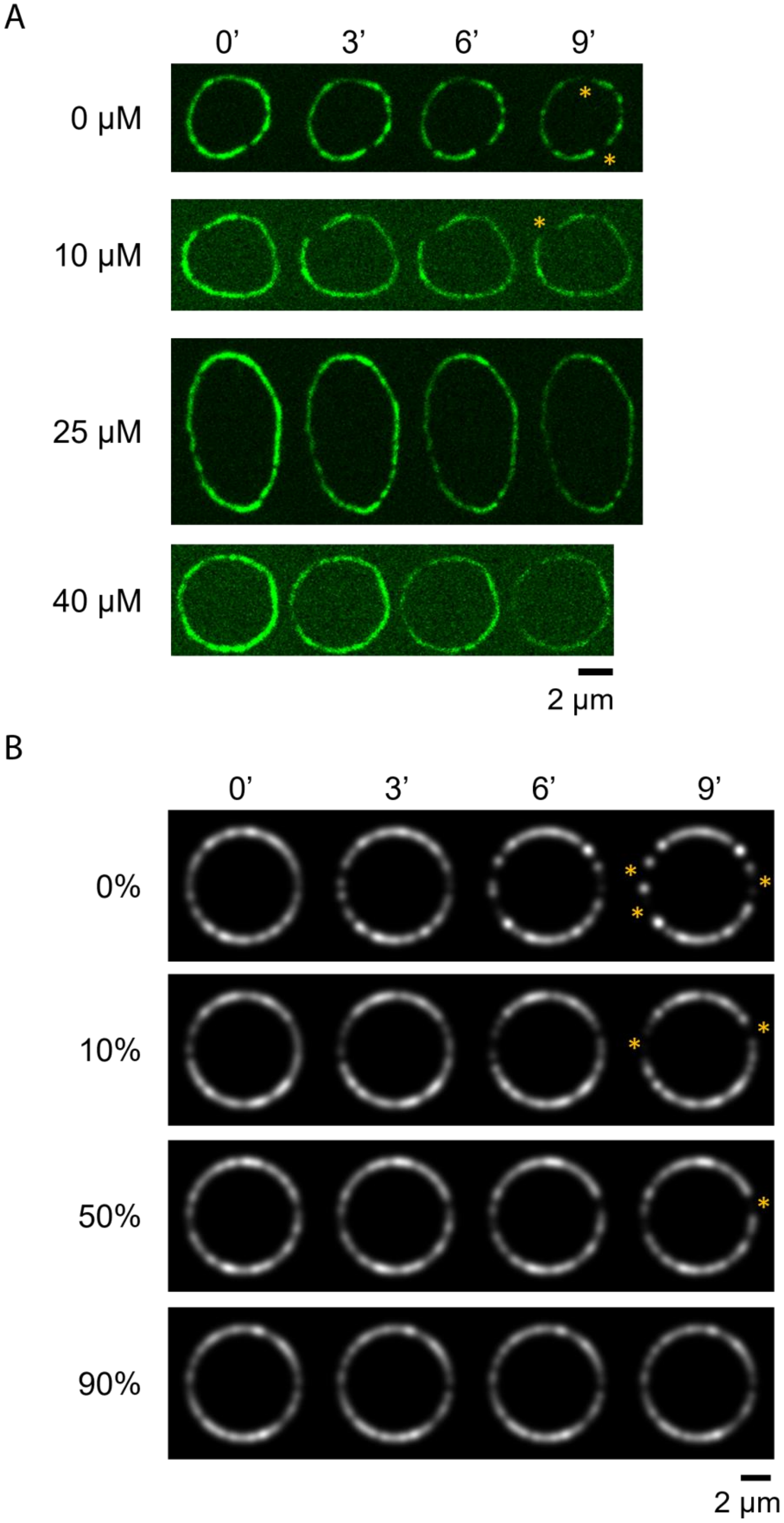

A
0’
3’
6’
9’
0 µM
10 µM
25 µM
40 µM
2 µm
B
0’
3’
6’
9’
0%
10%
50%
90%
2 µm

Fig. S6. In simulations and experiments hierarchical aggregation is progressively slowed down by increasing degrees of myosin II inhibition. (A) Time-lapse fluorescence micrographs of isolated rings in cell ghosts of *S. japonicus* labelled with Rlc1-GFP after addition of blebbistatin at the indicated concentrations followed by addition of 0.5 mM ATP. Images are max-intensity projections of rings from z-series data at the indicated times. Yellow asterisks indicate apparent gaps in myosin fluorescence of width 600 nm or greater after 9 min. Without blebbistatin myosin aggregates hierarchically, and with time increasing numbers of gaps of growing size are present (top row). The number and size of gaps is lower in the presence of blebbistatin. At higher blebbistatin levels, no gaps are seen after 9 min. (B) Simulated confocal fluorescence images of myosin-II distributions in cell ghosts at the indicated times with the indicated fractions of myosins deactivated. Prior to each simulation, an equilibration run lasting 11 min was performed to reach steady state (times $t < 0$). Turnover was then abolished and myosin deactivation was initiated for the simulation proper ($t \geq 0$). Yellow asterisks indicate myosin gaps > 600 nm after 9 min. At higher myosin deactivation levels, fewer gaps are present after 9 min.

Table S1. Key parameters values for simulations of rings in *S. Japonicus* cell ghosts.

<table>
<tr><th rowspan="2">Parameter</th><th rowspan="2">Meaning</th><th colspan="2">Value</th><th rowspan="2">Legend</th></tr>
<tr><th>in protoplasts</th><th>in cell ghosts</th></tr>
<tr><td>ρ_{for}</td><td>Initial mean formin dimer density</td><td colspan="2">$15\ \mu\mathrm{m}^{-1}$</td><td>(A)</td></tr>
<tr><td>ρ_{myo}</td><td>Initial mean myosin cluster density</td><td colspan="2">$18.75\ \mu\mathrm{m}^{-1}$</td><td>(B)</td></tr>
<tr><td>r_{sev}</td><td>Actin filament severing rate by cofilin</td><td>$1.8\ \mu\mathrm{m}^{-1}\mathrm{min}^{-1}$</td><td>$0.0409\ \mu\mathrm{m}^{-1}\mathrm{min}^{-1}$</td><td>(C)</td></tr>
<tr><td>$k_{\mathrm{off}}^{\mathrm{for}}$</td><td>Off rate of formins</td><td>$0.023\ \mathrm{s}^{-1}$</td><td>0</td><td>(D)</td></tr>
<tr><td>$k_{\mathrm{off}}^{\mathrm{myo}}$</td><td>Off rate of myosin clusters</td><td>$0.026\ \mathrm{s}^{-1}$</td><td>$2.2\ \times 10^{-4}\ \mathrm{s}^{-1}$</td><td>(E)</td></tr>
<tr><td>$k_{\mathrm{off}}^{\mathrm{x}}$</td><td>Off rate of α-actinin</td><td colspan="2">$3.3\ \mathrm{s}^{-1}$</td><td>(F)</td></tr>
<tr><td>f_{s}</td><td>Myosin stall force per filament</td><td colspan="2">$4\ \mathrm{pN}$</td><td>(G)</td></tr>
<tr><td>v_{myo}^{0}</td><td>Myosin load-free velocity</td><td colspan="2">$0.24\ \mu\mathrm{m/s}$</td><td>(H)</td></tr>
<tr><td>r_{myo}</td><td>Capture radius of myosin-actin binding</td><td colspan="2">$80\ \mathrm{nm}$</td><td>(I)</td></tr>
<tr><td>l_{p}</td><td>Persistence length of actin</td><td colspan="2">$10\ \mu\mathrm{m}$</td><td>(J)</td></tr>
<tr><td>γ_{myo}</td><td>Membrane anchor drag coefficient of myosin cluster</td><td colspan="2">$9.36\ \mathrm{nN}\cdot\mathrm{s}/\mu\mathrm{m}$</td><td>(K)</td></tr>
<tr><td>γ_{for}</td><td>Membrane anchor drag coefficient of formin dimer</td><td colspan="2">$1.9\ \mathrm{nN}\cdot\mathrm{s}/\mu\mathrm{m}$</td><td>(L)</td></tr>
</table>

(A) Ref. (1).

(B) Using 16 heavy chains per cluster, and the previously measured density of Myo2 myosin-II heavy chains (~3000 Myo2 heavy chains in a ~10 μm long ring, ref. (1)).

(C) Value in protoplasts is estimated in ref. (2). Value in cell ghosts is chosen such that at t = 40 min 60% of F-actin in the ring is lost compared to t = 0, consistent with previous experiments (3).

(D) Value in protoplasts is estimated from FRAP measurements of Cdc12p (4). In cell ghosts, actin loss in the presence of ATP and Phallacidin is negligible throughout constriction, suggesting formin molecules do not unbind the ring (3).

(E) Value in protoplasts is estimated from FRAP measurements of myosin light chain Cdc4p (5). In cell ghosts, myosin loss is set to be consistent with Fig. S3A.

(F) Obtained from ref. (6).

(G) Estimated in ref. (7) using node motions measured there.

(H) Estimated from ref. (8). See ref. (9) for details.

(I) The coiled coil tail of Myo2 is 65 nm long (10). The capture radius in the simulation is the tail length plus 15 nm to account for the length of the head and neck domain.

(J) Obtained from refs. (11), (12).

(K) Chosen so that the aggregation time observed in simulations and experiments in *S. japonicus* ghosts are similar.

(L) Estimated in ref. (2).

Movie S1 (separate file). Simulated cell ghost contractile ring of Fig. 3B at the indicated times. Turnover was abolished at t = 0. Formin dimer size and actin thickness not to scale. Scale bar: 1 μm.

SI References

1. J. Q. Wu, T. D. Pollard, Counting cytokinesis proteins globally and locally in fission yeast. *Science* **310**, 310-314 (2005).
2. M. R. Stachowiak *et al.*, Mechanism of cytokinetic contractile ring constriction in fission yeast. *Dev Cell* **29**, 547-561 (2014).
3. T. G. Chew *et al.*, Actin turnover maintains actin filament homeostasis during cytokinetic ring contraction. *J. Cell Biol.* **216**, 2657-2667 (2017).
4. A. Yonetani *et al.*, Regulation and targeting of the fission yeast formin cdc12p in cytokinesis. *Mol. Biol. Cell* **19**, 2208-2219 (2008).
5. R. J. Pelham, F. Chang, Actin dynamics in the contractile ring during cytokinesis in fission yeast. *Nature* **419**, 82-86 (2002).
6. Y. Li *et al.*, The F-actin bundler alpha-actinin Ain1 is tailored for ring assembly and constriction during cytokinesis in fission yeast. *Mol. Biol. Cell* **27**, 1821-1833 (2016).
7. M. R. Stachowiak *et al.*, Mechanism of cytokinetic contractile ring constriction in fission yeast. *Dev. Cell* **29**, 547-561 (2014).
8. B. C. Stark, T. E. Sladewski, L. W. Pollard, M. Lord, Tropomyosin and myosin-II cellular levels promote actomyosin ring assembly in fission yeast. *Mol. Biol. Cell* **21**, 989-1000 (2010).
9. S. Wang, B. O'Shaughnessy, Anchoring of actin to the plasma membrane enables tension production in the fission yeast cytokinetic ring. *Mol. Biol. Cell* **30**, 2053-2064 (2019).
10. M. Bezanilla, T. D. Pollard, Myosin-II tails confer unique functions in Schizosaccharomyces pombe: characterization of a novel myosin-II tail. *Molecular biology of the cell* **11**, 79-91 (2000).
11. D. Riveline, C. H. Wiggins, R. E. Goldstein, A. Ott, Elastohydrodynamic study of actin filaments using fluorescence microscopy. *Phys. Rev. E* **56**, R1330-R1333 (1997).
12. A. Ott, M. Magnasco, A. Simon, A. Libchaber, Measurement of the persistence length of polymerized actin using fluorescence microscopy. *Phys. Rev. E* **48**, R1642-R1645 (1993).
